# A National Pension Fund for Hospital Officials and Nurses

**Published:** 1887-04-02

**Authors:** 


### A National Pension Fund for Hospital Officials and Nurses.

### The Views of Officials, Sisters, Nurses, and others.

### By Pietro J. Michelli, Secretary, St. Mary's Hospital, Paddington.

The fact recorded in last week's Hospital, that only four nurses had sent in their names in support of a General Pension Fund, I attribute to the nurses not knowing anything about it. I don't think The Hospital often gets into the nurses' sitting-rooms, and if it does it is seldom read. Nurses are not newspaper readers, and when they have finished their day's work they seek other relaxation than the study of hospital matters. I am sure that this subject of the Pension Fund only wants to be brought to their notice, and you will hear from a great many. I would suggest that a small poster be printed, setting forth in as few words as possible the nature of the scheme, and that this be sent to the matrons of each hospital with a request that she will have it suspended in the nurses' sitting and dining rooms.

As to the scheme itself there can be no doubt but that it is excellent, the only thing being how to work it so as to meet the views of the majority. Suggestions have been made that the public should be asked to start the fund, but I think we ought to help ourselves. Without some sacrifice on the part of each we cannot expect to reap a benefit hereafter. I should like to see the fund started on the principle of the India Fund. Everyone joining pays a sum or sums per annum, but is not eligible to receive any benefit until after ten years' service; and not then, unless physically incapacitated. After twenty-five years' service the subscribers to the fund will enjoy the full pension.

I do not think that those who leave the hospital world before ten years' service should receive any benefit from the fund, unless they choose to make the annual payments, in which case they can become recipients as though they had not retired. The idea suggested by Mrs. Bedingfeld that next-of-kin should receive anything from the fund would not work. It is the payments of members who retire, or die early, that would enable the fund to give adequate assistance to those who have laboured long in the hospital or nursing world.

*** Our experience as to nurses not being newspaper readers does not confirm Mr. Michelli's views on the subject. Will other secretaries (metropolitan and provincial) send their views on this important subject for publication?—Ed.

### By a Trained Nurse willing to Subscribe.

The question has often occurred to me, How is it that among all the existing charitable societies there has not been, so far as I know, one established which provides a pension for trained nurses when they are unable to work? I am aware of the existence of the Trained Nurses' Annuity Fund, which grants a small amount when one has attained the age of fifty, including other conditions; but as the majority of women enter the profession at twenty-three, or even younger, I very much doubt if it would be possible continue in the work until fifty.

I have the opinion of many trained nurses, and they would only be too glad to subscribe to a fund which would ensure a pension for them when they could no longer earn for themselves. There is a good society which provides for sickness and death, but one cannot expect everything from one source. Why not put the subject before Miss Nightingale this Jubilee year? for she has the welfare of all nurses near at heart.

By Miss M. J. Thompson, Matron of the Oldham Infirmary.

Would you kindly insert this letter in your valuable Hospital? I am so anxious to help this poor nurse into a home or hospital, as she is entirely depending upon the help I can procure for her. The hospital has been defraying her expenses at the Home in New Brighton, and paying her salary for the last six weeks, but, of course, now as her medical attendant assures me she must give up work for several months, I am anxious to do something for her.

I think it is high time more provision was made for our nurses in hospitals; and surely, in this Jubilee year, nurses should receive some consideration, more especially when they are ill and disabled for work. I know of no home or institution for nurses. If you would kindly insert this letter I should be deeply grateful.

By a Private Nurse.

I beg to express my gratitude for the efforts made to establish a "National Annuity Fund" for nurses and others engaged in hospital work. I feel sure most nurses can subscribe £2 this year, £1 to be paid into the fund on joining, the other quarterly, and I venture to think that probationer nurses who are receiving salaries might subscribe five or ten shillings a year. Surely a nursing community of twelve to fifteen thousand people can and will co-operate to help each other in time of sickness and old age.

By another Private Nurse.

I am much interested in the scheme of setting on foot a "National Pension Fund for Hospital Trained Nurses." I think if some such scheme as Mrs. Bedingfeld suggests could be arranged, I feel sure nurses would be pleased indeed to subscribe. I should be glad to subscribe, not so much annually, but not less than a stated sum, and as much more as I could afford, which would depend upon my income, which varies; for I am a "private" nurse. And I know from experience that there are many "outsiders" who employ private nurses who would be pleased to send in a donation to the general fund, in recognition of the nurse's services at the termination of her case, especially as so many of the Institutions who send out private nurses, forbid the nurses accepting a present, and the public usually like to benefit the nurses who nurse them or their friends more than the institutions who supply them with nurses. I sincerely hope that some of the many nurses who I know approve of this scheme, will have the courage to write to you, and tell you so themselves.

*** The National Pension Fund will be open to all competent nurses, whether engaged in hospital or private nursing.—Ed. T. H.

